# 

**Published:** 1853-06

**Authors:** 


VOL. II. NEW SERIES. No. 2.

### THE NORTH-WESTERN MEDICAL AND SURGICAL JOURNAL:

PUBLISHED MONTHLY,

AT TWO DOLLARS PER ANNUM.

EDITED BY

W. B. HERRICK, M.D.,

PROFESSOR OF ANATOMY AND PHYSIOLOGY IN RUSH MEDICAL COLLEGE; SURGEON TO THE U. S. MARINE HOSPITAL, ONE OF THE SURGEONS OF THE ILLINOIS GENERAL HOSPITAL, ETC.

AND

H. A. JOHNSON, A.M., M.D.

**JUNE, 1853.**

CHICAGO:

BALLANTYNE & CO., PUBLISHERS & PRINTERS,

NEW POST-OFFICE BUILDINGS, COR. OF CLARK AND RANDOLPH-STS.,

OPPOSITE THE SHERMAN HOUSE.

1853.

VOL X. WHOLE SERIES. No. 2.

